# Amyloid particles facilitate surface-catalyzed cross-seeding by acting as promiscuous nanoparticles

**DOI:** 10.1073/pnas.2104148118

**Published:** 2021-08-30

**Authors:** Nadejda Koloteva-Levine, Liam D. Aubrey, Ricardo Marchante, Tracey J. Purton, Jennifer R. Hiscock, Mick F. Tuite, Wei-Feng Xue

**Affiliations:** ^a^Kent Fungal Group, School of Biosciences, University of Kent, CT2 7NJ Canterbury, United Kingdom;; ^b^School of Physical Sciences, University of Kent, CT2 7NJ Canterbury, United Kingdom

**Keywords:** protein aggregation and assembly, Sup35 yeast prion protein, amyloid β peptide, atomic force microscopy, *Saccharomyces cerevisiae*

## Abstract

Amyloid seeds are nanometer-sized protein particles that accelerate amyloid assembly as well as propagate and transmit the amyloid protein conformation associated with a wide range of protein misfolding diseases. However, seeded amyloid growth through templated elongation at fibril ends cannot explain the full range of molecular behaviors observed during cross-seeded formation of amyloid by heterologous seeds. Here, we demonstrate that amyloid seeds can accelerate amyloid formation via a surface catalysis mechanism without propagating the specific amyloid conformation associated with the seeds. This type of seeding mechanism is demonstrated through quantitative characterization of the cross-seeded assembly reactions involving two nonhomologous and unrelated proteins: the human Aβ42 peptide and the yeast prion–forming protein Sup35NM. Our results demonstrate experimental approaches to differentiate seeding by templated elongation from nontemplated amyloid seeding and rationalize the molecular mechanism of the cross-seeding phenomenon as a manifestation of the aberrant surface activities presented by amyloid seeds as nanoparticles.

## Significance

The formation of disease-associated fibrillar amyloid structures can be accelerated by preformed amyloid seeds. This seeding process is thought to occur solely through elongation at amyloid fibril ends, resulting in the templated propagation of the protein conformation encoded in the seeds. We demonstrate that amyloid seeding does not always proceed through templated elongation and show that amyloid seeds are nanoparticles that can accelerate the formation of new heterologous amyloid without templating the protein conformation encoded in the seeds. We provide experimentally testable criteria to distinguish seeding through a templated elongation mechanism from surface catalysis and present mechanistic insights into the amyloid seeding and cross-seeding phenomenon. These findings have wide implications for our understanding of the molecular basis of amyloid cross-interactions.

**Fig. 1.**
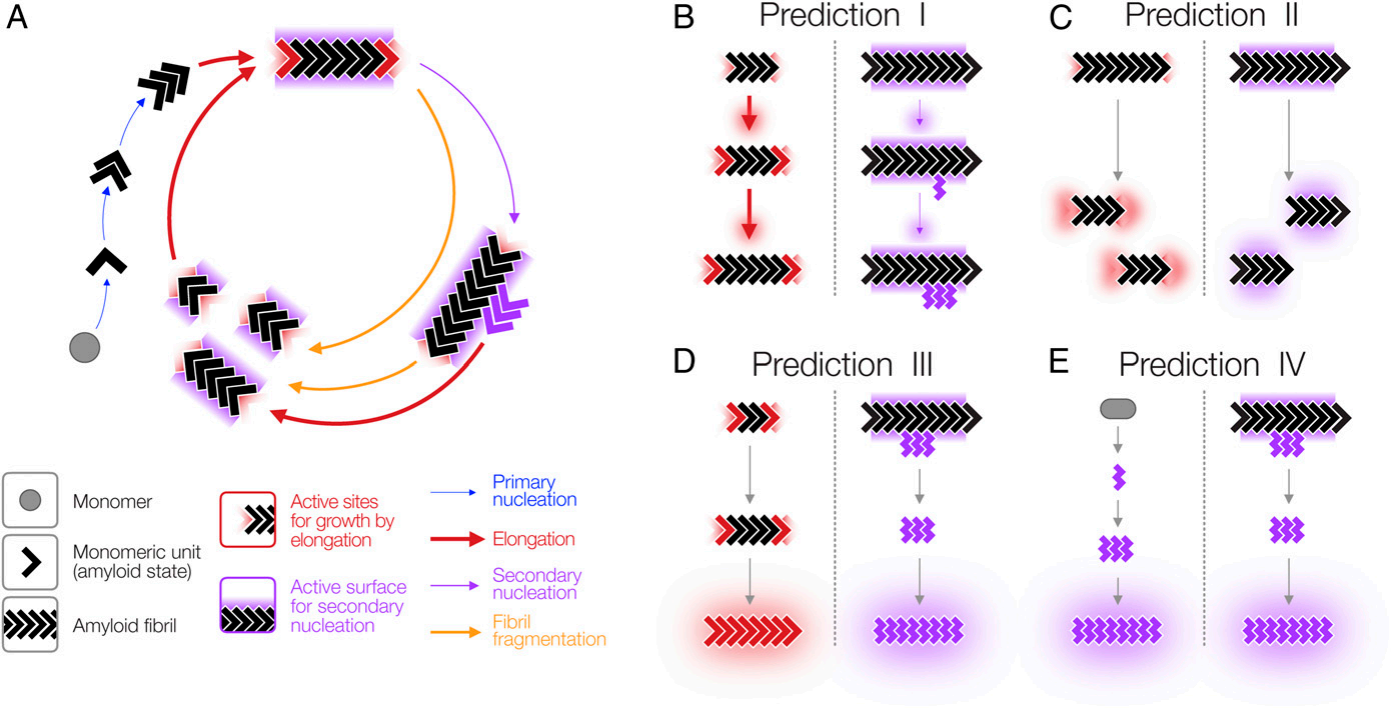
Schematic illustration of the molecular processes in the amyloid life cycle together with experimentally testable hypotheses. (*A*) The amyloid lifecycle with key sites and surfaces for templated growth and secondary nucleation highlighted in red and purple, respectively. (*B*–*E*) Comparison of a seeding mechanism based on templated growth at fibril ends and a seeding reaction with fibril growth promoted by a fibril surface-catalyzed nucleation mechanism. Experimentally testable and comparable features for each prediction are highlighted by red or purple glow. (*B*) Prediction I: A surface-catalyzed seeding reaction is nucleation dependent and, therefore, is slow, with a lag phase that cannot be readily eliminated by the seeds, compared to a faster seeding reaction through elongation. (*C*) Prediction II: The number of active sites for seeding through templated elongation (red) depends on the number of particles, while the number of active surfaces for surface-catalyzed seeding mechanism (purple) depends on the protein mass concentration. (*D*) Prediction III: The morphology of the fibrils newly formed by surface-catalyzed seeding does not need to be the same as that of the seeds. (*E*) Prediction IV: Indistinguishable fibril morphology and biological activity is produced from the same monomers under the same conditions independently of the seeds used for a surface-catalyzed seeding reaction. All arrows represent multiple dynamic and reversible steps along the lifecycle and the thickness of the arrows illustrate typical relative magnitudes of the rates involved in each of the processes.

Amyloid particles are associated with numerous neurodegenerative and/or age-related human disease such as Alzheimer’s disease, Huntington’s disease, Parkinson’s disease, and type 2 diabetes mellitus (1, 2). The slow nucleation-dependent process that converts normally soluble protein or peptide precursors into their amyloid conformation (3) can be bypassed through the addition of preformed amyloid particles, the seeds. This phenomenon, which effectively accelerates amyloid growth and propagates the amyloid conformation, is called seeding. The seeded growth of amyloid, as well as transmissible forms of amyloid known as prions, via the templated addition of monomer units or small oligomers to the ends of preformed fibril seeds (4–6) is known as fibril elongation (Fig. 1*A*).

Elongation at amyloid fibril ends has long been viewed as the sole mechanism of seeded amyloid growth, in which the specific amyloid conformation encoded in the seeds is propagated upon the addition of new monomers or small oligomers at fibril ends (4, 7). This mechanistic assumption has been, for example, applied in attempts to propagate patient-derived amyloid material for structural studies and has been challenged through evidence to suggest that the structure of the seeds does not necessarily propagate though seeded growth (8). Furthermore, a protein can form amyloid in an accelerated manner upon addition of amyloid seeds preformed with precursors of very different or even completely nonhomologous amino acid sequences (9). The molecular mechanism of this phenomenon, often termed “cross-seeding,” remains unresolved, because the current models for the fibril elongation growth mechanism cannot explain the full range of molecular behaviors observed during amyloid cross-seeding.

For mammalian disease-associated amyloidogenic proteins, cross-seeding activity may be a key process promoting a synergy between amyloid associated disorders. A number of studies have demonstrated that two different amyloidogenic disorders may arise in the same individual and, in so doing, impact on the respective occurrence and pathologies of the disorders (10, 11). For example, it has been proposed that cross-seeding between Aβ and α-synuclein (12) and Aβ42 and IAPP (13) might contribute the observed statistical correlations between the occurrence of Alzheimer’s disease and Parkinson’s disease or type 2 diabetes, respectively (14–16). Thus, understanding the fundamental nature of the molecular crosstalk between amyloidogenic disease-associated proteins and the underlying molecular mechanism will provide essential clues to a better understanding of how these diseases originate, propagate, and even transmit between individuals.

Amyloid fibrils are protein filaments with monomeric units arranged in the characteristic cross-beta molecular architecture held together by noncovalent interactions and hydrogen bonds parallel to the fibril axis (17). The fibrils are usually in the order of 10 nm in width, and amyloid seeds are typically small amyloid fibril fragments often less than 100 nm in length. Thus, amyloid seeds are bona fide nanoparticles (i.e., particulate materials with individual particle dimensions in the order of or below 100 nm for at least two out of three spatial directions) (18). Like any type of nanoparticle, the small sizes of amyloid seeds confer these particles with high surface-to-volume ratios. These surfaces are frequently capable of interacting with molecules in their surroundings and can be coated with a corona of proteins and other macromolecules in addition to ions and small molecules in biological environments (19, 20).

The surfaces of amyloid seeds contain active sites where growth by elongation takes place at their termini, as well as surfaces parallel to the cross-beta hydrogen bonds that have previously been shown to be exceptionally active in catalyzing heterogeneous nucleation of new amyloid in what is called “secondary nucleation” as is observed in the formation of Aβ amyloid (21). Thus, amyloid seeds, in addition to promoting the propagation of the specific amyloid conformation encoded by the monomeric units at their elongation active sites through templated growth at fibril ends, may also be able to catalyze generic surface-mediated assembly like any nanoparticle and accelerate the formation of heterologous amyloid through catalyzing heterogeneous nucleation.

In order to test whether such a general surface-catalyzed mechanism can explain and rationalize the molecular mechanism of amyloid cross-seeding and to show that the seeding and templating activities of amyloid seeds can potentially be mechanistically uncoupled, we investigated the cross-seeding interactions between two unrelated amyloidogenic proteins: human Aβ42 that is associated with Alzheimer disease (2) and the amyloid-forming protein Sup35NM that is a component of a prion-based epigenetic switch in the yeast *Saccharomyces cerevisiae* (7). We have chosen these two proteins, because being from two organisms at different ends of the evolutionary spectrum, they do not coexist in the same biological context. Sup35 (eRF3) is present in human cells but lacks the N-terminal and middle (NM) regions critical for amyloid formation and propagation (7). Furthermore, Aβ and Sup35NM have low sequence similarities (*SI Appendix*, Fig. S1 *A* and *C*) and dissimilar amino acid compositions (*SI Appendix*, Fig. S1*B*) as expected from two functionally unrelated proteins. The amyloid aggregation mechanism of human Aβ42 has been studied in considerable detail (21), revealing an assembly mechanism dominated by secondary nucleation accelerated by preexisting fibril surfaces. Sup35, on the other hand, is regarded as a functional amyloid, and its assembly mechanism reveals a strong component of templated elongation (4, 7). Here we demonstrate that these two unrelated proteins are capable of cross-seeding each other (i.e., the presence of the amyloid seed of one protein is capable of accelerating amyloid formation of the other protein despite their dissimilar sequence, structure, and biological origins). We also show that these heterologous interactions are “asymmetric” (22) (i.e., the kinetic effect of the seeds is different with respect to amyloid assembly of each other). We demonstrate that these cross-seeding interactions are mass sensitive but not particle number sensitive. In addition, by exploiting the well-characterized prion phenotype [*PSI*^+^] associated with the amyloid state of Sup35 protein, we demonstrate the phenotypic outcome on cells propagating either the self-seeded or cross-seeded Sup35NM prion particles in vivo. Together, our in vitro and in vivo results demonstrate that amyloid seeds are nanoparticles with active surfaces that can mediate the cross-seeding of heterologous amyloid through generic surface-catalyzed reactions, resulting in accelerated amyloid growth, without structural templating of the precise amyloid conformation encoded in the seeds.

## Results

### Both Templated Elongation and Surface-Catalyzed Nucleation Mechanisms Can Promote Accelerated Amyloid Assembly, but Each Produce Different Seeding Behaviors.

Seeding is a process defined as the acceleration of amyloid formation in the presence of seeds. To test whether amyloid seeds are nanoparticles that can accelerate the formation of new heterologous amyloid through nontemplated surface-catalyzed assembly reactions, we first examined in detail the mechanistic differences between templated seeding reactions by elongation versus the nontemplated surface-catalyzed seeding reactions (Fig. 1 *B*–*E*). In so doing, we sought to establish whether the addition of seeds can accelerate amyloid assembly either through templated growth by elongation at fibril ends (left schematic in Fig. 1 *B*–*E*) or surface-catalyzed nucleation of new amyloid (right schematic in Fig. 1 *B*–*E*) and if these two pathways can be distinguished experimentally. As illustrated in Fig. 1 *B*–*E*, if given amyloid particles are capable of seeding or cross-seeding the formation of new amyloid through a surface-catalyzed nucleation mechanism instead of templated elongation, one would predict a number of differences in the molecular and kinetic behaviors of the seeded reactions that should be distinguishable experimentally.

Firstly, the presence of active surfaces should reduce but not eliminate the nucleation barrier for assembly (Fig. 1*B*, prediction I), since surface-catalyzed assembly would still be a nucleation-dependent process. Consequently, such a reaction would still go through a slow nucleation phase, albeit faster compared to nucleation in the absence of surface catalysis (*SI Appendix*). Thus, the addition of seeds active in surface catalysis of nucleation would only be capable of reducing the length of the lag phase but not eliminate the lag phase entirely as would be observed with seeding reactions that proceed through templated elongation. Secondly, the number of growth active sites for templated elongation is only present at fibril ends and therefore relates to the particle concentration of the seed particles. On the other hand, surfaces along the seeds that potentially can catalyze heterogeneous nucleation such as secondary nucleation or surface-catalyzed seeding events should depend on the total length of the particles, which is in turn proportional to the mass or monomer equivalent concentration of seeds and not the number concentration of the seed particles (*SI Appendix* and Fig. 1*C*, prediction II). Thirdly, if new amyloid is formed through seeding by a surface-catalysis mechanism, then the fibril morphology of the newly formed fibrils does not need to be the same as the morphology of the seeds (Fig. 1*D*, prediction III). Finally, if newly formed amyloid assembles through seed surface-catalyzed reactions, then their morphology and the biological response they elicit should only be linked to their monomer precursors and the conditions applied but not the seeds (Fig. 1*E*, prediction IV). These four experimentally testable differences were therefore used to rationalize whether amyloid particles can act as broad-spectrum seeds that are capable of accelerating the formation of new and heterologous amyloid primarily due to the activities of their surfaces in the same way as the action of nanoparticles.

### Self-Seeded Growth of Both Aβ42 and Sup35NM Amyloid Fibrils Proceeds through Templated Fibril Elongation.

To characterize the heterologous seeding potential of amyloid particles, we chose to investigate the self-seeding and cross-seeding interactions between two unrelated proteins: human Aβ42 and yeast Sup35NM. These two amyloid-forming proteins are native to different organisms, do not naturally coexist in the same biological context, and do not share any known evolutionary linkages. Furthermore, human Aβ is associated with Alzheimer’s disease known to be statistically correlated with the occurrence of a number of other amyloid-associated diseases (14–16), while the full-length Sup35 protein can become a transmissible prion and can be regarded as a functional amyloid (7). These two proteins also have low sequence similarities (*SI Appendix*, Fig. S1*C*) and are dissimilar in terms of size, charge, amino acid composition (*SI Appendix*, Fig. S1), and fibril structures. Therefore, each of these two proteins should not be able to grow onto fibril seeds preformed by the other protein through templated assembly. Hence, they provide ideal and unbiased tests of the heterologous seeding capabilities of amyloid particles.

**Fig. 2.**
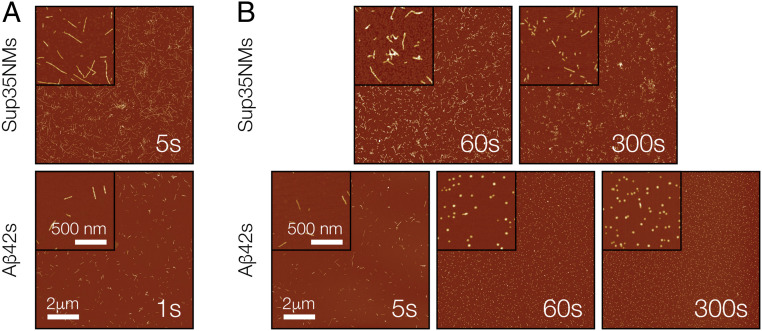
AFM images of Aβ42s and Sup35NMs fibril seeds. (*A*) AFM images of initial samples of Aβ42s and Sup35NMs seeds after brief controlled sonication to disperse the fibrils. (*B*) AFM images of Aβ42s and Sup35NMs seeds after a different length of controlled sonication. For all images, the insets show 4 times–magnified areas for each image. (Scale bars, 2 μm and 500 nm.) The length of sonication is indicated in the lower right corner in all images.

We first generated fibrillar seed particles of Aβ42 and Sup35NM (here called Aβ42s and Sup35NMs, respectively) in vitro by incubating the monomeric precursors of respective amyloid (here called Aβ42m and Sup35NMm, respectively) under common fibril growth solution conditions as both proteins form amyloid fibrils under physiological pH. To generate Aβ42s fibril particles, a synthetic Aβ42 peptide (Bachem, Germany) was used. The peptide samples were dissolved in 6M GdnHCl at pH10, and Aβ42m monomers were purified by gel filtration using a Superdex 75 column immediately prior to assembly to ensure the generation of reproducible and high-quality Aβ42s amyloid seeds (23, 24). Monomeric Sup35NMm protein was produced recombinantly in *Escherichia coli* and assembled as described previously (25). Aβ42s and Sup35NMs fibrils were subsequently dispersed by brief controlled sonication (*Materials and Methods*) and imaged using atomic force microscopy (AFM). Interestingly, the Aβ42s particles were apparently less resistant to sonication compared to Sup35NMs particles. Consequently, 1 s of controlled sonication was sufficient to disperse the Aβ42s fibrils for imaging, while at least 5 s of controlled sonication was required to disperse Sup35NMs fibrils prior to imaging. Fig. 2*A* shows AFM images of Aβ42s and Sup35NMs fibril seeds imaged by AFM.

Controlled sonication is a method commonly used to fragment amyloid fibrils for seed generation. To validate controlled sonication as being capable of generating Aβ42s and Sup35NMs seed samples with different particle concentrations, while retaining their respective original mass concentrations, the seed samples were subjected to controlled sonication for different periods of time. As shown in Fig. 2*B*, for Sup35NMs, increasing the sonication time from 5 s to 300 s decreased the lengths of the seed particles and therefore increased the particle concentration as expected and previously seen (25). The same effect of decay in particle lengths and a rise in particle concentration as sonication time increased from 1 s to 60 s was also seen for Aβ42s particles (Fig. 2*B*). As previously observed, the Aβ42s particles were less resistant to sonication compared to Sup35NMs particles, with 60 s of controlled sonication generating a large number of small nanoparticles less than 100 nm as seen using AFM (Fig. 2*B*). Additional sonication did not further alter their size distribution noticeably as would be expected due to their already small sizes (26, 27). To further confirm the quality the Aβ42s and Sup35NMs seed samples, dynamic light scattering (DLS) was performed on these seed samples after controlled sonication (*Materials and* *Methods*). As shown in *SI Appendix*, Fig. S2, the DLS experiments show that both the Aβ42s and Sup35NMs seed samples consisted of a distinct distribution of fragmented fibrils without the presence of any major secondary particle distributions. Thus, the AFM and DLS experiments together confirmed the presence of high-quality seed samples for both Aβ42s and Sup35NMs formed under the same solution conditions.

**Fig. 3.**
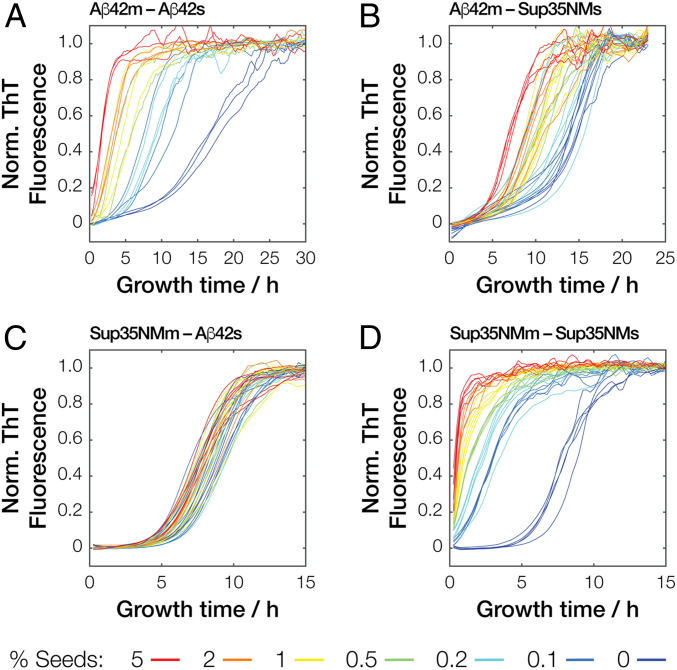
Kinetics traces of seeded amyloid formation monitored by ThT fluorescence. Typical normalized traces of (*A*) monomers of Aβ42 (Aβ42m) self-seeded by Aβ42 seeds (Aβ42s) or (*B*) by Sup35NM seeds (Sup35NMs) as well as (*C*) Sup35NM monomers (Sup35NMm) seeded by Aβ42 seeds (Aβ42s) or (*D*) self-seeded by Sup35NM seeds (Sup35NMs). For each monomer–seed combination, at least nine replicate reaction traces from three independent experiments containing reactions with different ratios (0 to 5% mol/mol) of seeds to monomer ratio added were collected, and three to five replicate traces are shown for each reaction for clarity. Both Aβ42s and Sup35NMs used were sonicated for 60 s.

**Fig. 4.**
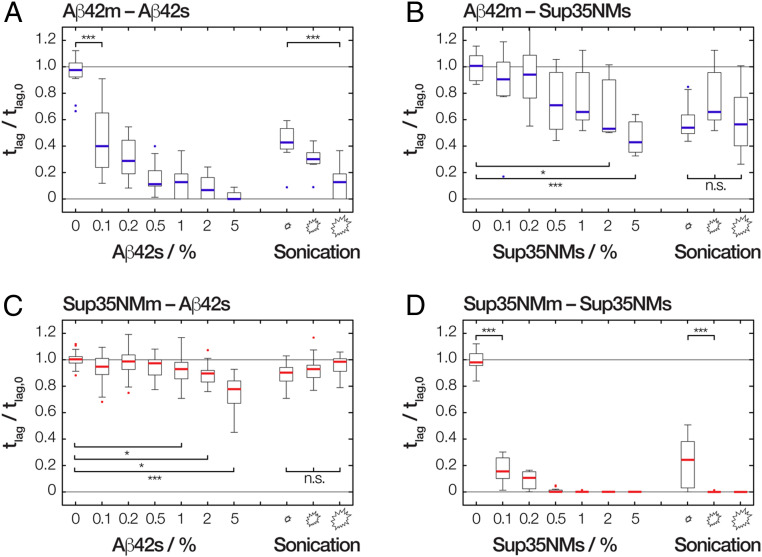
The relative reduction in the length of lag phase upon addition of seeds compared to unseeded amyloid formation. The relative length of the lag phase (*t*_lag_) values of (*A*) Aβ42 monomers (Aβ42m) self-seeded by Aβ42s or (*B*) by Sup35NMs as well as (*C*) Sup35NM monomers (Sup35NMm) seeded by Aβ42s or (*D*) self-seeded by Sup35NMs seeds are shown as ratio to *t*_lag_ values of respective unseeded reactions (*t*_lag,0_). For each protein pair, seeding reactions were performed with varying concentration of seeds sonicated to 60 s as well as varying degrees of sonication at 1% seeds added as indicated by varying sized symbols (small, medium, and large sonication symbols denote for Aβ42s 1 s, 5 s, and 60 or 300 s of sonication, and for Sup35NMs, 5 s, 60 s, and 300 s of sonication, respectively). The *t*_lag_ values were extracted from the kinetics traces using the method illustrated in *SI Appendix*, Fig. S3. The distribution of *t*_lag_ values for each experiment are shown as box plots with the thick line representing the median, and each bar represents the data from at least nine replicate reactions from three independent experiments. One-way ANOVA with Tukey's Honestly Significant Difference Procedure was carried out for statistical multiple pair-wise comparison. The “*”, “***”, and “n.s.” labels denote when the sample mean relative *t*_lag_ values of the compared pairs are significantly different with *P* value less than 0.05, *P* value less than 0.001, or not significant with *P* value more than 0.05, respectively.

**Fig. 5.**
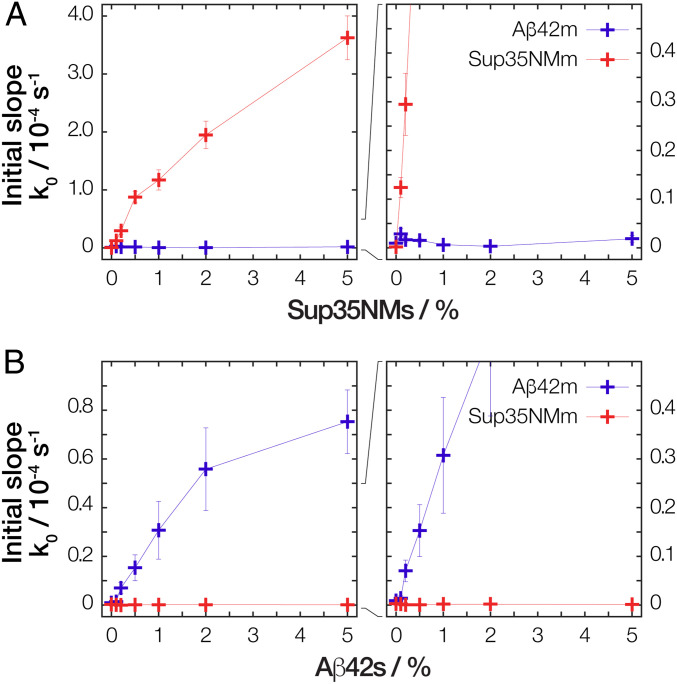
The increase of the initial slope of the kinetic traces as function of increasing percentage of seeds. The average initial slope values (*k*_0_) extracted from the ThT traces (Fig. 3) of (*A*) Aβ42 monomers (Aβ42m) or Sup35NM monomers (Sup35NMm) seeded by Sup35NMs seeds as well as (*B*) seeded by Aβ42s seeds are shown as “+” with error bars representing the SE of mean. The *k*_0_ values were extracted from the kinetics traces using the method illustrated in *SI Appendix*, Fig. S3. The error bars for some reactions with low *k*_0_ values are not visible due to being smaller than the symbol.

To confirm the ability of Aβ42s and Sup35NMs particles to seed the formation of new amyloid, we performed a series of seeded fibril growth kinetic assays monitored using the fluorescence of the amyloid-specific Thioflavin T (ThT) dye in a 96-well plate format, involving both Aβ42m and Sup35NMm each seeded by seeds formed from monomers of the same sequence (i.e., self-seeding). The kinetic profiles of amyloid growth were mapped as a function of low seed concentrations ranging from 0.1 to 5% to determine the parameters of respective seeded growth and their dependence on seed particle concentration (Fig. 3 *A* and *D*). Subsequently, we extracted and analyzed two parameters characteristic for the amyloid assembly kinetics (*SI Appendix*, Fig. S3); the length of the lag phase (*t*_lag_, compared in Fig. 4 *A*–*D*) and the initial slope (*k*_0_, compared in Fig. 5 *A* and *B*) from each reaction trace. For Sup35NM fibril seeds (Sup35NMs) self-seeded with Sup35NM monomers (Sup35NMm), the presence of 0.1 to 5% monomer molar equivalent seeds in a growth reaction with 10 μM total monomer equivalent concentration dramatically shortened or eliminated the lag-phase in all cases. Importantly, as seen in Fig. 4*D*, as little as 0.5 to 1% monomer molar equivalent of the seeds was sufficient to eliminate the lag phase by reducing *t*_lag_ to 0 h. This behavior is entirely consistent with templated monomer addition to the preformed fibril-seed ends acting as a dominant mechanism of the elongation growth (4, 7). Seeding efficiency also increased with higher added particle concentrations either through increased monomer molar equivalent seeds or through increased sonication at the same monomer molar equivalent (Fig. 4*D*). As seen in Fig. 5*A* (red lines), the initial slope of seeded growth curves is directly proportional to particle concentration, which in turn is proportional to the number of active growth sites for elongation at fibril-ends at low particle concentrations. At high concentrations, the elongation process can become saturated as also seen in other amyloid systems (e.g., ref. 28). Global analysis of the kinetic traces (*SI Appendix*, Fig. S4*D* and Table S1) further confirmed that a model describing a mechanism dominated by elongation fitted the data. Therefore, the data demonstrate that the self-seeded growth of Sup35NM proceeds through templated elongation at fibril ends as expected (4).

Similarly to Sup35NM, assembly of Aβ42 monomers (Aβ42m) was significantly accelerated by the addition of as little as 0.1% preformed Aβ42 fibril-seeds (Aβ42s). The lag-phase was eliminated in the presence of as low as ∼1% monomer molar equivalents or more of seeds (Fig. 4*A*). Increasing the seed particle concentration also increased the initial slope of the growth reactions in the same linear manner at low seed particle concentrations as seen with Sup35NM and other amyloid-forming systems (Fig. 5*B*, blue lines). Furthermore, increasing the particle concentration at constant molar equivalent monomer concentration of seeds by sonication also shortened the lag-phase, demonstrating that the seeding reaction is dependent on the particle concentration of the seeds (Fig. 4*A*). As with self-seeded Sup35NMm assembly, global analysis of the kinetic traces (*SI Appendix*, Fig. S4*A* and Table S1) confirmed that a model describing a mechanism dominated by elongation also fitted the data of self-seeded Aβ42m assembly. In this case, the globally fitted model shows a minor systematic deviation when compared to the experimental nonseeded kinetic traces (dark blue traces in Fig. 3*A*) that displayed a shallow slope in their lag phase baselines. This is likely due to residual Aβ42 aggregates in the monomer protein fraction used at the start of the experiment (29). However, the globally fitted model with an elongation-dominated mechanism fully reproduced the kinetic effect of seeding in eliminating the lag phase at the low-seed concentrations used. Therefore, the self-seeded growth of Aβ42 amyloid also displayed all the hallmarks of a seeding mechanism dominated by templated elongation at fibril-seed ends.

### Growth of Aβ42 Amyloid Fibrils Is Accelerated by Sup35NM Seeds in a Mass Concentration–Dependent, but Not Particle Number–Dependent, Manner.

Having established the self-seeded reactions proceed through templated elongation at fibril-seed ends for both Aβ42 and Sup35NM, we next investigated whether the seeds formed from these two unrelated amyloidogenic proteins are able to accelerate the amyloid forming reaction of each other in cross-seeded reactions. First, we investigated the growth kinetics of Aβ42m assembly in the presence of Sup35NMs seeds. In a series of fibril growth kinetic assays monitored using ThT fluorescence (Fig. 3*B*), the addition of heterologous seeds (in this case, the unrelated Sup35NMs) in low concentrations (5% or less monomer molar equivalents) to monomer solutions of Aβ42 was able to statistically significantly reduce the lag time of the Aβ42 amyloid formation (Fig. 4*B*). These experiments show that Sup35NMs can act as seeds that accelerate amyloid formation of Aβ42, albeit with less efficiency in reducing the duration of the lag phase than Aβ42s. Interestingly, the addition of 5% monomer molar equivalents of Sup35NMs failed to eliminate the lag phase of Aβ42 amyloid growth (*t*_lag_ > 0 in Fig. 4*B* and *k*_0_ ∼ 0 in Fig. 5*A*, blue lines). This finding is not consistent with a templated elongation mechanism but is consistent with Sup35NMs being nanoparticles that act as generic seeds through surface-catalyzed heterogeneous nucleation, because the slow nucleation events should still occur (Fig. 1, prediction I). Global analysis of these cross-seeded kinetic traces (*SI Appendix*, Fig. S4B) also confirm that the data are consistent with a surface-catalyzed heterogeneous nucleation mechanism.

If the nucleation events catalyzed by the large available surface area of Sup35NMs (and not active growth sites at fibril-ends) directs the shortening of the lag-phase in these heterologous cross-seeded reactions, then the number of free ends that are responsible for the templating process should exert no significant effect on the efficiency of seeding (Fig. 1, prediction II) as long as the monomer equivalent concentration (equivalent to the mass concentration of seeds) is maintained. We tested this prediction by adding Sup35NMs sonicated to different extents and therefore should have identical monomer mass concentration but different seed particle concentrations (25) to the Aβ42m solutions (Fig. 2*B*). Remarkably, adding an identical mass of Sup35NMs seeds that were subjected to less time of sonication did not significantly increase the length of lag-phase of Aβ42 assembly, nor did the length of lag phase decrease significantly when identical mass of Sup35NMs seeds, which were subjected to a greater sonication time, were added. Thus, changing the particle concentration of Sup35NMs while maintaining identical protein mass concentration of seeds produced no significant effects on the length of lag-phase of Aβ42 fibril forming reactions (Fig. 4*B*). These results provided kinetic evidence to support that Sup35NMs, while biologically and structurally unrelated to Aβ42s, are able to accelerate the amyloid formation of Aβ42 by acting as nanoparticles that provide their surface for catalyzing Aβ42 amyloid formation.

### Growth of Sup35NM Amyloid Fibrils Is Also Accelerated by Aβ42 Seeds but Not to the Same Extent.

We next tested whether Aβ42s were also able to accelerate the amyloid assembly of Sup35NMm in the opposite heterologous cross-seeded reaction. As before, we incubated Sup35NMm solutions in the presence of various amounts of Aβ42s and monitored the fibril growth kinetic using ThT fluorescence (Fig. 3*C* and *SI Appendix*, Fig. S4*C*). For Sup35NM amyloid formation, Aβ42s was also able to significantly shorten, but not eliminate, the length of the lag phase. However, this lag phase-shortening effect was reduced in magnitude compared with the other three reaction pairs analyzed, with around a 25% reduction in the length of the lag phase upon addition of 5% monomer molar equivalents of Aβ42s compared to unseeded reactions (Figs. 4*C* and 5*B*, red lines). Similar to the effect of Sup35s on Aβ42m assembly, a change in particle concentration without a change in monomer equivalent mass concentration of Aβ42s by varying sonication time for Aβ42s led to no significant effect on the length of the lag phase of Sup35NMm assembly. These experiments indicate that Aβ42s particle surfaces are also able to accelerate the nucleation of Sup35NM amyloid fibrils, but the efficiency is less compared to the effect of Sup35NMs surfaces on Aβ42m assembly (compare Fig. 4 *B* and *C*). This asymmetry is consistent with the fact that the efficiency of surface-catalyzed heterogeneous nucleation mechanism for cross-seeding is dependent on the physical and chemical properties of the seed surfaces in the same way as any nanoparticle interaction with biology.

### Sup35NM Amyloid Formed through Self-Seeding and Cross-Seeding with Aβ42s Seeds Display Indistinguishable Fibril Morphology and Induce Identical Prion Phenotypes In Vivo.

**Fig. 6.**
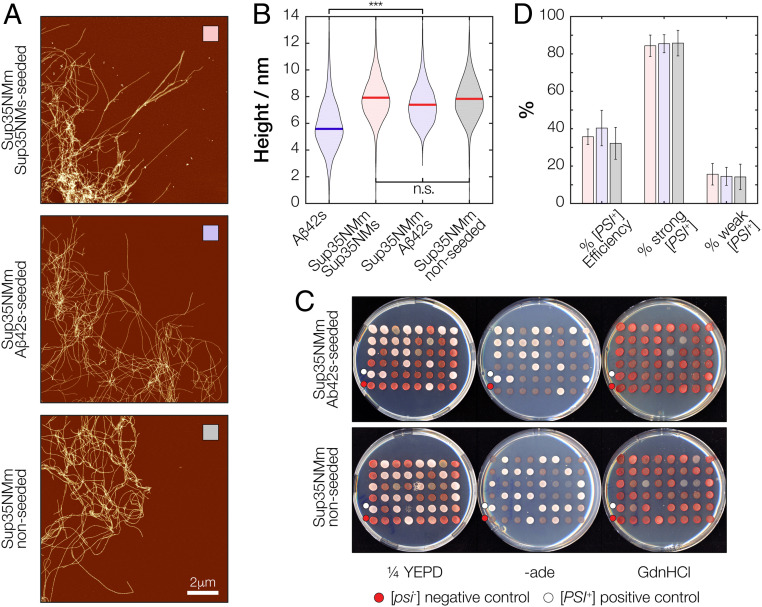
Sup35NM amyloid fibrils formed through self-seeding, cross-seeding using Aβ42s seeds, or unseeded reactions are morphologically and biologically indistinguishable. (*A*) Typical AFM images of Sup35NM fibrils formed through self-seeding (*Upper*), cross-seeding using Aβ42s (*Middle*), or unseeded (*Lower*) reactions. Scale bar indicates 2 μm in all three images. (*B*) The distribution of fibril heights, characteristic of their widths, for the Sup35NM fibrils were extracted from the images in *A* and represented as violin plots. The thick line in each distribution represents the mean height. The “***” and “n.s.” labels denote when the mean height values of the compared sample pairs are significantly different as indicated by one-way ANOVA with Tukey's Honestly Significant Difference Procedure in which *P* value is less than 0.001 or not significant with *P* value more than 0.05, respectively. (*C*) Efficiency of the fibrils shown in *A* in conferring yeast cells with the [*PSI*^*+*^] prion phenotype upon transfection. Yeast cells transfected with the fibrils were replica plated onto 1/4YEPD and -ade synthetic media to check for the [*PSI*^*+*^] prion phenotype and 1/4YEPD supplemented with 3 mM GdnHCl to eliminate any false positives. The fibrils were sonicated for 600 s before transfection experiments. Negative [*psi*^*−*^] and positive [*PSI*^*+*^] phenotype controls are indicated on the plate images with red and white dots, respectively. (*D*) Quantification and comparison of the transfection efficiency and the [*PSI*^*+*^] phenotype displayed by the yeast cells transfected with the fibrils shown in *A*. The bars indicate average values of at least three independent experiments performed on separate days, and the error bars represent the SE of mean.

If the mechanism of heterologous cross-seeding involves generic seeding through heterogeneous nucleation catalyzed by surfaces of seeds as nanoparticles, then the amyloid fibril morphology of the newly grown amyloid does not need to be the same as that of the seeds (Fig. 1, prediction III). The morphology and the biological properties of the newly grown amyloid may also be the same under the same solution conditions and independently of whether they were formed through homologous self-seeding or heterologous cross-seeding (Fig. 1, prediction IV). To test these structural predictions, we used AFM to analyze Sup35NM fibrils formed in two seeded reactions, either self-seeded with preformed Sup35NMs fibrils or cross-seeded with Aβ42s fibrils (Fig. 6*A*). The morphology of Sup35NM fibrils grown by cross-seeding with Aβ42s (Fig. 6*A*), characterized by the height distribution (Fig. 6*B*), was strikingly different compared to that of Aβ42s. The fibril heights of Aβ42-seeded Sup35NM fibrils were significantly different to those of the Aβ42s but indistinguishable to self-seeded or de novo grown Sup35NM fibrils. This is consistent with the specific amyloid conformation of Aβ42s seeds not being imposed on the newly formed Sup35NM fibrils, confirming the structural prediction III (Fig. 1*D*, prediction III). Importantly, because fibrils in these heterologous cross-seeded reactions were indistinguishable from those formed from self-seeding with Sup35NMs or from de novo assembly of Sup35NMm (Fig. 6*A*), and the height distributions for all these samples were also not significantly different from each other (Fig. 6*B*), these fibril populations must be formed mainly due to the monomer sequence and the solution condition, in agreement with the structural prediction IV (Fig. 1*E*, prediction IV). Taken together, these results support the hypothesis that the heterologous seeds in this case merely accelerated the kinetics of amyloid formation without passing on its precise conformation by a lack of templating.

Similar to the mammalian prion protein PrP that can exist in structurally different prion conformation and distinct “strains” (30), the yeast [*PSI*^*+*^] prion linked to the amyloid form of the Sup35 protein can also exist as different conformational “strains” generating phenotypically distinct but stable [*PSI*^*+*^] “variants” (31). The phenotype linked to a given [*PSI*^*+*^] variant (i.e., a defect in translation termination leading to stop codon read-through) reflects the strength of the underlying biological prion activity and is based on the different mechanical and structural properties of the Sup35 assemblies in that variant (31–33). The phenotype imposed by a [*PSI*^*+*^] variant can be readily detected and visualized using a well-established colorimetric assay utilizing *S. cerevisiae* strains carrying the *ade1-14* nonsense mutant allele. In these *ade1-14* yeast strains, the presence of the [*PSI*^*+*^] prion leads to suppression of the *ade1-14*-linked phenotype (i.e., red colonies requiring adenine [Ade]) to restore the wild-type Ade^+^ phenotype with colony color ranging from pink to white depending on the specific [*PSI*^*+*^] variant (31, 34, 35). Thus, the [*PSI*^*+*^] phenotype provides a sensitive in vivo test of prediction IV (Fig. 1*E*, prediction IV, i.e., whether fibrils formed from self-seeding with Sup35NMs were comparable to those formed by heterologous cross-seeded reactions through generic surface catalyzed action of Aβ42s seeds as nanoparticles).

Nonseeded (i.e., de novo grown from Sup35NMm), self-seeded, and Aβ42s cross-seeded Sup35NM particles were introduced into spheroplasts of red colony forming prion-free [*psi*^*−*^] yeast cells by protein transfection (*Materials and Methods* and ref. 25). The phenotypes of the resulting transfected colonies were initially assessed by plating onto a rich growth medium (1/4YEPD; *Materials and Methods*) and onto a defined medium lacking adenine. To establish whether white Ade^+^ transfectants induced by each of the introduced amyloid samples contained the [*PSI*^*+*^] prion, these colonies were then replica-plated onto 1/4YEPD containing 3 mM GdnHCl. At this low concentration, GdnHCl inhibits [*PSI*^*+*^] propagation leading to a loss of the prion form and restoration of the [*psi*^*−*^] red, Ade^−^ phenotype (Fig. 6*C*). Protein transfection of a [*psi*^*−*^] yeast strain with the various Sup35NM amyloid particles, either grown from different seeds or no seeds, resulted in the generation of indistinguishable number and phenotypical pattern of [*PSI*^*+*^] transfectants, with the majority showing a “strong” white Ade^+^ phenotype while the remaining 15% had a “weak” [*PSI*^*+*^] phenotype (i.e., pink or dark pink Ade^+^ colonies) (Fig. 6*D*). These in vivo studies support the conclusion that the fibril particles have an indistinguishable morphology independent of the seeds they were exposed to and that they also give rise to the same biological phenotype, which in turn is sensitive to small conformational difference in the amyloid architecture (32, 36). Therefore, the seeding of Sup35NMm monomers with heterologous Aβ42s amyloid seeds did not generate amyloid particles that affect the conformation of Sup35, which was faithfully transmitted and propagated in vivo. This finding agrees with the structural predictions III and IV (Fig. 1 *D* and *E*, predictions III and IV) for a cross-seeding mechanism involving generic heterogeneous nucleation catalyzed by surfaces of seeds as nanoparticles.

### Aβ Amyloid Fibril Seeds Are Capable of Inducing [*PSI*^*+*^] Phenotype upon Transfection into Yeast Cells.

**Fig. 7.**
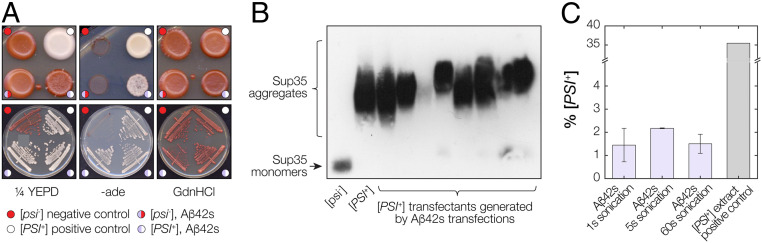
Transfection of yeast cells with Aβ42s results in enhanced [*PSI*^*+*^] conversion. (*A*) A typical positively converted colony by Aβ42s is shown together with positive [*PSI*^*+*^] and negative [*psi*^*−*^] phenotype controls on replica plated as well as streaked plates. The cells were plated onto 1/4YEPD and -ade synthetic media to check for the [*PSI*^*+*^] prion phenotype and 1/4YEPD supplemented with 3 mM GdnHCl to eliminate any false positives. The identity of the colonies is indicated by the colored dots. (*B*) SDD-AGE analysis followed by immunoblotting revealed that aggregates of Sup35 were formed in the yeast cells converted to [*PSI*^*+*^] by Aβ42s transfection. Cell extract of positive [*PSI*^*+*^] and negative [*psi*^*−*^] controls is shown in the left two lanes for comparison. (*C*) Quantification and comparison of the transfection efficiency displayed by the yeast cells transfected with Aβ42s seed particles at different particle concentrations produced by sonication, with the light purple bars indicating average values of at least three independent experiments performed on separate days with the error bars represent the SE of mean. The right dark gray bar indicates the transfection efficiency displayed by the yeast cells transfected with a cell extract of positive [*PSI*^*+*^] control (*SI Appendix*, Fig. S5) for comparison.

The in vitro kinetics studies show that the surfaces of Aβ42s seeds can interact and cross-seed the formation of Sup35NM amyloid fibrils by acting as nanoparticles that promote surface-catalyzed interactions (Figs. 3*C*, 4*C*, and 5*B*). To test whether heterologous Aβ42s nanoparticles introduced into a [*psi*^*−*^] yeast cell can trigger the appearance of the Sup35-based [*PSI*^*+*^] prion in vivo, we next transfected yeast spheroplasts prepared from a [*psi*^*−*^] *ade1-14* yeast strain with the in vitro–assembled Aβ42s particles. Approximately 2% of the Aβ42s-transfected colonies analyzed contained a mixture of red and white colony-forming cells (Fig. 7*A*) suggestive of a low rate of prion conversion in the primary transfected cell. When these mixed red/white colony-forming transfectants were restreaked onto fresh 1/4YEPD medium, the rare white colonies that formed displayed all of the properties associated with the presence of the [*PSI*^*+*^] prion (i.e., a stable white colony phenotype on 1/4YEPD medium; growth on selective medium lacking adenine; and stable loss of the white phenotype when grown in the presence of 3 mM GdnHCl) (37). Since the presence of minuscule amounts of Aβ42s seeds cannot be readily detected due to the slow conversion rate, the large dilution factor when single particles enter the cell volume, and when the cell goes on to divide for many cycles to form colonies, control experiments were carried out to confirm conversion to [*PSI*^*+*^] by Aβ42s transfection. Firstly, semidenaturing agarose gel electrophoresis (SDD-AGE) gel assay followed by immunoblotting were carried out, confirming that the Sup35 protein were indeed converted to an aggregated state in all tested [*PSI*^*+*^] cells converted by Aβ42s transfection (Fig. 7*B*), with the same pattern compared to Sup35 in cells converted by Sup35NMs seeds (25). Secondly, the efficiency of transfection and subsequent conversion to [*PSI*^*+*^] was much less frequent using Aβ42s than transfection with Sup35NMs particles (25, 31) or in vivo–formed Sup35 particles (*SI Appendix*, Fig. S5), but the samples with increasing particle concentration generated through controlled sonication of Aβ42s did not significantly increase their efficiency in conversion of cells to [*PSI*^*+*^] (Fig. 7*C*), consistent with prediction II (Fig. 1*C*). Finally, a control experiment was carried out to compare the low frequency of [*PSI*^*+*^] occurrence following transfection by Aβ42s with the frequency of the spontaneous de novo formation of [*PSI*^*+*^] in the absence of any amyloid seeds. The results show that the frequency of [*PSI*^*+*^] occurrence was around 1000-fold higher in yeast cells following transfection by Aβ42s compared to spontaneous de novo formation of [*PSI*^*+*^] in nontransfected [*psi*^*−*^] cells (Table 1).

**Table 1.** Frequency of [*PSI*^*+*^] appearance for yeast cells transfected with Aβ42s compared with de novo formation of [*PSI*^*+*^] in wild-type *S. cerevisiae* strains

Strain	Frequency of [*PSI*^*+*^] appearance*	95% CI
*74D-694 transfected with Aβ42s* ^†^	2.2 × 10^−2^	1.5 × 10^−2^–2.9 × 10^−2^
*74D-694 Haploid* ^†^	1.1 × 10^−5^	0.1 × 10^−5^–2.1 × 10^−5^
*74D-694 Diploid* ^†^	2.4 × 10^−5^	1.5 × 10^−5^–3.2 × 10^−5^
*243/6a* ^‡^	10^−7^–10^−5^	—
*74D-694* ^§^	5.8 × 10^−7^	4.6 × 10^−7^–7.5 × 10^−7^

* Median values in [*psi*^*−*^][*PIN*^*+*^] yeast cells.

^†^ This study.

^‡^ (61).

^§^ (62).

To confirm that amyloid seeds are capable of acting as promiscuous nano-particles that can promote enhanced heterologous amyloid formation in vitro and in vivo and this is not limited to Aβ42s, we tested whether amyloid seed particles formed from Aβ40 (Aβ40s) possess the same type of seeding capabilities toward Sup35 in yeast cells, despite the structures of Aβ40 amyloid is very different to that of Aβ42 amyloid (e.g., refs. 38–40). Analysis of Sup35NMm amyloid forming reactions seeded with in vitro formed Aβ40s (*SI Appendix*, Fig. S6*A*) confirmed that Aβ40s is indeed also capable of increasing the rate of Sup35NM amyloid formation in vitro (*SI Appendix*, Fig. S6*B*). Importantly, yeast transfection experiments carried out using Aβ40s seed samples demonstrate that they are also able to enhance [*PSI*^*+*^] conversion of cells in vivo when transfected into [*psi*^*−*^] yeast cells (*SI Appendix*, Fig. S6C), with a lower frequency compared with in vivo formed Sup35 aggregates but comparable to that seen with Aβ42s.

Taken together, these in vivo analyses show that Aβ40s and Aβ42s particles can increase the appearance of the [*PSI*^*+*^] prion in vivo when introduced into prion-free [*psi*^*−*^] yeast cells despite no evident biological or structural links existing between the yeast Sup35 protein, its maintenance chaperone network in vivo, and Aβ sequences. These findings are consistent with the promotion of nonnative heterologous surface interactions by Aβ amyloid particles in vivo as we also observed in vitro and demonstrate that heterologous cross-seeding of amyloid may reflect the generic property of amyloid seeds and their surfaces in biological systems.

## Discussion

Synergetic heterologous interactions of amyloid aggregates, as exemplified by amyloid cross-seeding, is a well-known phenomenon and frequently studied in relation to human diseases linked to human amyloidogenic proteins (reviewed in ref. 9). In these cases, cross-seeding has been assumed to also contribute to why some amyloid-associated diseases coincide with the formation of other nonhomologous amyloid aggregates. However, the molecular mechanism of how such cross-seeding processes proceed in vitro or in vivo remain unresolved because the widely accepted templated elongation model does not explain how completely different sequences and structures are capable of templating each other. In addition, the importance of primary sequence similarity for the observed cross-seeding between heterologous protein aggregates is not clear (e.g., refs. 41, 42). Thus, the current view of cross-seeding through templated elongation alone does not readily explain the synergetic statistical links between amyloid diseases associated with nonhomologous proteins (14–16). Here, under rigorously controlled conditions, we have investigated the cross-seeding interactions between two amyloid-forming proteins, namely, human Aβ42 and yeast Sup35NM. These two proteins were chosen because they are entirely unrelated in terms of sequence, structure, and biological function and consequently should not be able to template the elongation growth of each other. Yet, we observed cross-seeding effects between these two proteins, in which nonhomologous fibrillar seeds significantly shortened the lag phase of amyloid-forming reactions, albeit to a much lesser extent compared to homologous seeds. We also found that the effect of nonhomologous cross-seeding in the fibril-forming reaction for these two proteins was not symmetric and instead depended on the aggregation properties of monomers under the reaction conditions used (43) and on the specific type of seeds used. In addition, in vivo studies whereby amyloid seeds can be introduced into a genetically marked strain of *S. cerevisiae* that reports the ability of those seeds to trigger the formation of Sup35 amyloid (31) were carried out. Whereas different in vitro–generated or extract-purified variants of Sup35NM fibrils when transfected into such yeast cells are faithfully propagated, creating different ratios of weak or strong [*PSI*^+^] phenotypes (31, 33, 44), Sup35NM fibrils formed in Aβ42-seeded reaction generated a mixture of [*PSI*^+^] phenotypes that were indistinguishable to those arising when de novo–formed or self-seeded Sup35NM fibrils generated in vitro were used (Fig. 6).

**Fig. 8.**
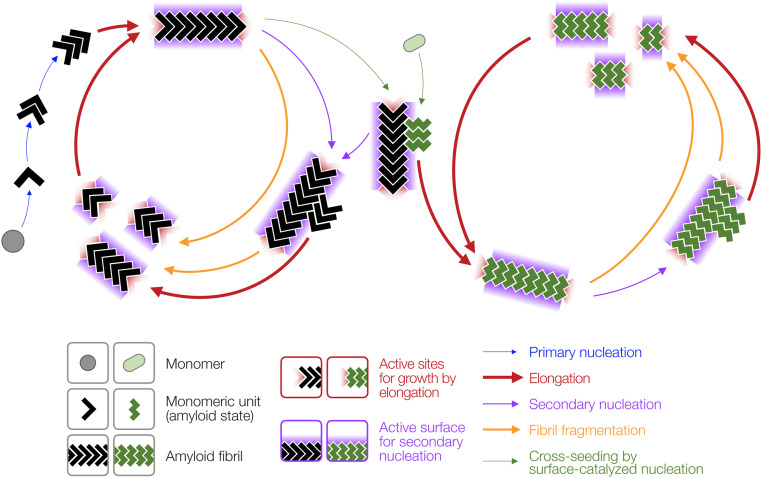
Schematic illustration of a cross-seeding mechanism involving surface-catalyzed heterogeneous nucleation by amyloid particles acting as promiscuous nanoparticles with active surfaces that also promote secondary nucleation. The surface-catalyzed cross-seeding is represented by the green arrows that links the lifecycles of two otherwise unrelated amyloid systems represented as black and green monomeric units, respectively. Sites for templated growth and surfaces for secondary nucleation or cross-seeding by surface-catalyzed nucleation are highlighted in red and purple, respectively. All arrows represent dynamic and reversible steps along the lifecycle and the thickness of the arrows illustrate typical relative magnitudes of the rates involved in each of the processes.

The results we obtained for the heterologous seeding action between these two unrelated amyloidogenic proteins are not consistent with templated elongation but are entirely consistent with the amyloid seeds acting as nanoparticles that affect heterologous amyloid assembly through surface-based interactions. In our experiments, both the Aβ42s and Sup35NMs seeds acted to accelerate the amyloid formation of the other protein, giving rise to the observed cross-seeding effect without acting as templates of their own conformation (Fig. 8). However, not all amyloid seeds are likely to be capable of accelerating amyloid formation for all amyloid sequences. For amyloid-forming protein pairs that do cross-interact, the balance between the templated fibril elongation at the fibril ends and the surface-catalyzed fibril formation pathways is likely dependent on factors such as the sequence similarity between the seed and the monomers, the conformational flexibility of the monomers, the solution conditions, and the surface properties of individual amyloid seeds. Indeed, heterologous surface catalysis is often utilized in organic synthesis while amyloid seeding by surface-catalyzed heterogeneous nucleation or retardation by surface interactions are commonly observed effects of polymeric and nonpolymeric nanoparticles alike (45).

Since nucleated protein assembly catalyzed by seed surfaces is not dictated by the amyloid conformation of the seeds in the same way as in templated elongation reactions, the resulting amyloid fibrils from surface-catalyzed reactions could have structures distinct from that of the seeds (Fig. 1, prediction III). Furthermore, the resulting amyloid structures could be diverse in their morphology. This is because seeding and cross-seeding through surface-catalyzed nucleation events may be expected to introduce heterogeneity in the resulting amyloid aggregates depending on the assembly conditions, whereas seeding though templating will propagate specific amyloid conformations and thereby reducing possible heterogeneity. For amyloidogenic proteins involved in misfolded protein diseases, heterologous amyloid particles would potentially allow the formation of a pallet of conformational variants or strains, with different levels of toxicity and infectious potential (46, 47). Thus, structural polymorphism (48–50) as a consequence of species heterogeneity modified by the cross-seeded reaction could lead to the generation of new toxic conformers, and their propagation could play an important role in disease.

Previous studies have demonstrated that Aβ42 aggregation is accelerated by an auto-catalyzed nucleation process. In common with the cross-seeding mechanism we address here, this autocatalytic process of secondary nucleation is a surface-driven process (21) and is one of the major processes involved in homologous amyloid assembly (Fig. 1). Thus, the surfaces of preformed amyloid seeds of Aβ42, even outside of active elongation sites at fibril ends (51), appear to be particularly active and capable of accelerating formation of new amyloid through promoting surface interactions. The secondary nucleation mechanism can accelerate amyloid formation as well as to generate small oligomeric species that could be biologically active in driving the toxic potential of Aβ42 amyloid (21). This suggests that the surfaces of small Aβ42 amyloid seeds, as nanoparticle surfaces, may be able to act as general amyloid formation catalysts for both homologous and heterologous sequences and, in the process of accelerating heterologous amyloid formation, generate some species that may possess cytotoxic potential. Interestingly, recent research has shown that for another yeast prion-forming protein Ure2, the surface-catalyzed secondary nucleation process does not dominate homologous assembly in presence of preformed fibrils (52). It has been suggested that the absence of secondary nucleation results in a reduced generation of toxic oligomeric species and therefore lower toxicity associated with formation and propagation of yeast prions. In contrast, a secondary nucleation mechanism has been inferred in cases involved in the amyloid formation of peptides and proteins associated with neurodegenerative diseases and type 2 diabetes (e.g., Aβ42, Aβ40, a-synuclein, IAPP, and insulin; reviewed in ref. 53). Thus, the generation of such amyloidogenic species with heightened toxic potential may depend on the surface properties of the seed particles and how these surfaces interact with both homologous and heterologous monomeric protein sequences that are present in the same biological milieu.

The enhanced likelihood of generating the Sup35-based [*PSI*^*+*^] prion upon transfection of prion-free [*psi*^*−*^] cells with Aβ42 fibril particles is consistent with our hypothesis that amyloid seeds as nanoparticles can influence the formation of heterologous amyloid via aberrant surface interactions. The presence of aberrant surfaces such as those presented by Aβ42 fibril particles in yeast cells may also act through modulating cellular proteostasis. This could therefore subtly affect the balance of proteins such as the molecular chaperones critical for the prion generation and propagation pathways (e.g., the AAA^+^-ATPase Hsp104) (54–56). Thus, Aβ42 fibril particles in vivo may provide surface-mediated interactions accelerating the formation and the propagation of the amyloid state in the cells though direct as well as indirect modes of action. These insights bring to the fore surface properties and surface interactions of amyloid particles as a key mesoscopic property to target in order to understand the origin of the amyloid cytotoxic potential and the synergetic link between different amyloid diseases as well as designing therapies to combat the disease processes associated with toxic amyloid.

## Materials and Methods

### Terminology.

Fibrillar seed particles of Aβ42 and Sup35NM used in the experiments are referred to as Aβ42s and Sup35NMs, respectively. The monomeric precursors of respective amyloid used in the experiments are referred to as Aβ42m and Sup35NMm, respectively. The terms Aβ42 and Sup35NM are used to refer to general aspects of the proteins or to the assembly of the respective amyloid.

### Protein Expression and Purification.

Sup35NM protein samples were produced as described previously (25) with minor changes as follows. The DNA sequence encoding the N-terminal NM region of the yeast Sup35 protein (residues 1 through 254) was amplified from plasmid pUKC1620 by PCR and cloned into pET15b as a *Bam*HI-*Nde*I fragment, resulting in an N-terminal His_6_-tag fusion protein. The resulting plasmid (pET15b-His_6_-NM) was then transformed into the *E. coli* strain BL21 DE3 (*F– ompT gal dcm lon hsdSB(rB- mB-) λ(DE3 [lacI lacUV5-T7 gene 1 ind1 sam7 nin5]*). For protein expression, this *E. coli* strain was grown overnight in 50 mL Lysogeny broth (LB) supplemented with 0.1 mg/mL ampicillin and then transferred to 1-L cultures of the same medium. On reaching an optical density at 600 nm (OD_600_) of ∼0.5, expression was induced by the addition of isopropyl ß-D-1-thiogalactopyranoside (1 mM final concentration) for 4 h. Cells were harvested at 6,000 rpm, and the cell pellets washed once in buffer A1 (20 mM Tris⋅HCl pH 8.0, 1 M NaCl, and 20 mM imidazole). Cells were pelleted again and the pellets kept at −80 °C for later use. For the affinity purification step, buffer A2 (20 mM Tris⋅HCl pH 8.0, 1 M NaCl, 20 mM Imidazole, and 6 M GdnHCl) was added to frozen cell pellets at a 5:1 (vol/vol) ratio, followed by sonication at an amplitude of 22 microns until the cell pellet was completely disrupted. This solution was then spun down at 13,000 rpm for 30 min and the supernatant collected. A total of 2 mL of Chelating Sepharose Fast Flow (GE Healthcare) was added to a small plastic column and prepared for affinity purification by sequential washing with 1 column volume (CV) of water, 0.2 M NiCl_2_, buffer A1, and buffer A2. The equilibrated resin was then resuspended in buffer A2 and added to previously collected supernatant. This mixture was then incubated for 1 h at room temperature with agitation to improve protein binding to the affinity resin. Centrifugation at 5,000 rpm was subsequently used to collect the resin, which was then washed in 5 mL buffer A2, resuspended in buffer A2, and transferred back to the column. After one wash with 1 CV buffer A2, elution was achieved by addition of 3 mL buffer A3 (20 mM Tris⋅HCl pH 8.0, 1 M NaCl, 0.25 M imidazole, and 6 M GdnHCl). The resulting eluate was immediately used for size-exclusion purification, which was run using a HiLoad 16/600 Superdex 200 pg (GE Healthcare) column in an ÄKTA Prime Plus chromatography system (GE Healthcare). The eluate was injected into the size-exclusion column previously equilibrated with 1 CV water followed by 1 CV buffer S1 (20 mM Tris⋅HCl pH 8.0, and 0.5 M NaCl) and 1 CV buffer S2 (20 mM Tris⋅HCl pH 8.0, 0.5 M NaCl, and 6 M GdnHCl). The relevant Sup35NM protein fractions were collected according to the A_280_ displayed throughout the run, diluted to 20 μM in buffer S2, and immediately used in fibril-forming reactions.

The Amyloid Beta (1-42) peptide (Aβ42) and Amyloid Beta (1-40) peptide (Aβ40) was purchased in 5-mg batches from Bachem (Germany). This was aliquoted in 0.5-mg stock batches and frozen at −20 °C. Monomers were further purified as described previously (23, 24) with minor modifications. Briefly, the Aβ42 was purified using gel filtration as follows: 0.5 to 1 mg of Aβ42 was dissolved in 1 mL 6 M GdnHCl. This was loaded onto a Superdex 75 10/300 GL column pre-equilibrated with 2 CV of 20 mM sodium phosphate, pH 7.4, and 0.01% NaN_3_ (buffer E). The monomer peak was eluted with buffer E and put on ice. The concentration was then determined using ultraviolet spectroscopy (280 nm) and adjusted to working concentration of 10 μM with buffer E before immediately proceeding with fibril-forming reactions. The Aβ40 was purified using gel filtration using the same procedure.

### Fibril Formation and Monitoring.

For Sup35NM fibril formation, 2.5 mL of 20 μM purified Sup35NM were buffer-exchanged into Fibril Forming Buffer (FFB; 20 mM sodium phosphate buffer pH 7.4, 50 mM NaCl) using a PD-10 column (GE Healthcare) as per the manufacturer’s instructions. Unless otherwise specified, protein concentration was measured using A280 and then adjusted to 10 μM using FFB. Protein samples were aliquoted into Protein LoBind tubes (Eppendorf) and polymerized at 30 °C quiescently for at least 48 h. For monitoring polymerization, 100-μL samples of protein were aliquoted into black low-binding hydrograde 96-well plates (BRAND), and ThT was added to a final concentration of 10 μM. The plate was sealed with Starseal Advanced Polyolefin Film (Starlab), and kinetics were monitored in a 96-well format (3) using a FLUOstar OMEGA plate reader (BMG Labtech) quiescently at 30 °C.

For Aβ42 and Aβ40 fibrils assembly reaction, a 10-μM solution of monomers purified as described above was either aliquoted either into Protein LoBind tubes or into black hydrograde 96-well plates (BRAND) with 10 μM of ThT for kinetic monitoring using identical method as described above for Sup35NM.

The fibril assembly reaction traces were analyzed in Matlab. At least nine replicate reaction traces from three independent experiments performed using independent protein samples prepared on different days were collected. For each trace, the length of the lag phase (*t*_lag_) and the initial slop (k_0_), both indicative of early processes of seeded amyloid assembly reactions, were extracted using the method illustrated in *SI Appendix*, Fig. S3. For the analysis of the *t*_lag_ values, one-way ANOVA with Tukey's Honestly Significant Difference Procedure was carried out for statistical multiple pair-wise comparison to determine whether the length of the lag phase was significantly different or not to unseeded reactions. Global analysis was carried out with numerical solutions of *SI Appendix*, Eq. **S4** obtained using ordinary differential equation solvers in Matlab. Uncertainties in the fitted kinetic parameters were estimated using a Bootstrap method with 500 resampled datasets.

### Fibril Fragmentation.

Fibril fragmentation was achieved by sonication over different periods of time using a probe sonicator (Qsonica Q125) at 20% amplitude in consecutive 5-s on/off cycles on an ice-cooled water bath.

### DLS.

All vials, tubes, and cuvettes used for preparing the samples were clean dry. All solvents used were filtered to remove any particulates that may interfere with the results obtained. The Aβ42 or Sup35NM fibril seed samples obtained after controlled sonication were diluted 10 times using the same FFB as in the fibril formation experiments. The samples were subsequently characterized by DLS at 25 °C using an Anton Paar Litesizer 500 instrument and the data processed using KalliopeTM Professional.

### Yeast Transfection with Amyloid Fibrils.

For yeast transfection with Sup35NM synthetic amyloid fibrils, a [*psi*^−^] derivative of the yeast strain 74D-694 (*MATα ade1-14 trp1-289 his3Δ-200 ura3-52 leu2-3,112*), and [*PIN*^*+*^] derivative of the same strain was used for transfections of Aβ42 fibrils. The transfection procedure was as previously described (25). Briefly, cells freshly grown in YEPD to an OD_600_ of 0.5 were washed, resuspended in 12 mL ST buffer (1 M sorbitol, 10 mM Tris⋅HCl pH 7.5), and spheroplasts were prepared by addition of 600 U of lyticase (Sigma L4025) and 10 mM DTT during incubation at 30 °C with agitation for 45 min. Spheroplasts were then harvested by centrifugation (400 *g*, 5 min), washed with 1.2 M sorbitol and STC buffer (1.2 M Sorbitol, 10 mM Tris⋅HCl pH 7.5, and 10 mM CaCl_2_), and then resuspended in 1 mL STC buffer. Premixture of 2 μL (∼1 μg) of plasmid DNA (pRS416), 10 μL single-stranded DNA (10 mg/mL), and 10 μL of freshly sonicated amyloid fibrils (described in the *Fibril Fragmentation* section) were combined with 100 μL spheroplast suspension for each transformation reaction. This transformation mix was then incubated for 10 min at room temperature, and then 0.9 mL of polyethylene glycol buffer (40% PEG 4000, 10 mM Tris⋅HCl pH 7.5, and 10 mM CaCl_2_) was added to each transformation. After 30 min at room temperature, the spheroplasts were collected by centrifugation (400 *g*, 5 min), resuspended in 200 μL SOS media (1 M Sorbitol, 25% YEPD, 10mM CaCl2), and added to sterile Top agar (-uracil synthetic complete media with 2% agar and 1.2 M Sorbitol) being kept at 48 °C, gently mixed, and then poured into agar plates previously prepared using the same media. Cells were allowed to grow for 3 to 4 d at 30 °C, and then colonies were individually picked into 96-well plates containing YEPD (yeast extract 1%, bactopeptone 2%, glucose 2%, and agar 2%). These were grown overnight at 30 °C with agitation and then replica plated onto 1/4YEPD (0.25%, bactopeptone 2%, glucose 2%, and agar 2%) and -ade synthetic media to check for the [*PSI*^*+*^] prion phenotype and 1/4YEPD supplemented with 3 mM GdnHCl to eliminate any false positives as 3 mM GdnHCl eliminates the [*PSI*^*+*^] prion (37). Fragmented amyloid fibrils used in transfection experiments were simultaneously prepared for imaging analysis using AFM as described in the *AFM Analysis* section. [*PSI*^*+*^] cells arising from transfections with Aβ42 fibrils typically generated colonies consisting of a mixture of white wand red cells. In such cases, the white colonies were subcloned on 1/4YEPD and -ade plates and rechecked for their [*PSI*^*+*^] prion phenotype on 1/4YEPD+GdnHCl plates.

### Preparation of Cell Extracts and SDD-AGE.

Cell extracts were prepared by first harvesting yeast cells (∼2 × 10^7^ cells) and resuspending the pellet in 100 μL PEB buffer (25 mM Tris⋅HCl pH 7.5, 50 mM KCl, 10 mM MgCl2, 1 mM EDTA, and EDTA-free Protease Inhibitor Mixture [Roche]). Approximately 1 pellet volume of small glass beads was added to the resuspended cells and lysis performed by vortexing at 4 °C. The lysate was then cleared by centrifugation (8,000 rpm, 10 min, 4 °C), and total protein concentration in the collected clear lysate was measured at A280. SDD-AGE analysis was performed as previously described (57). Briefly, ∼100 μg total protein were loaded per lane. Samples were loaded in a 1.5% agarose gel prepared in buffer G (20 mM Tris, 200 mM glycine) and ran in Laemmli buffer (20 mM Tris, 200 mM glycine, and 0.1% SDS). Proteins were transferred using semidry blotting and transfer buffer T (20 mM Tris, 200 mM Glycine, 0.1% SDS, and 15% [vol/vol] methanol) onto a polyvinylidene difluoride (PVDF) membrane for 90 min at 10 V. The anti-Sup35 primary antibody MT50 was used in Western blot analysis as described previously (25).

### Quantification of Spontaneous De Novo Appearance Frequency of [*PSI*^*+*^].

The frequency of spontaneous de novo conversion of yeast cells from [*psi*^*−*^] to [*PSI*^*+*^] was quantified using an adaptation of a previously published protocol (58). Here, the [*psi*^*−*^][*PIN*^*+*^] derivative of the strain 74-D694 was used. A total of 10 independent and freshly grown colonies were randomly selected from YEPD plates and grown in YEPD media to reach an OD_600_ of 0.5. The cells were then washed, resuspended in sterile water, and plated on -ade medium at three dilutions (around 10^5^ cells/plate, 10^4^ cells/plate, and 10^3^ cells/plate) to ensure that the residual growth on -ade plates does not affect the appearance of Ade^+^ colonies. To determine the concentration of viable cells, an aliquot of each culture was used for a serial dilution plated on YEPD medium. Ade^+^ colonies were counted on -ade plates after 10 d of growth at 25 °C. To confirm [*PSI*^*+*^] phenotype, the Ade^+^ colonies were replica-plated on GdnHCl media and characterized further by SDD-AGE for confirming the presence of SDS-resistant Sup35 aggregates. For the Aβ42-transfected cells, colonies selected on -ura plates 3 d after transfection were grown overnight in YEPD media and stamped on –ade, 1/4YEPD, and 1/4YEPD media containing 3 mM GdnHCl. [*PSI*^*+*^] formation rates were calculated according to the formula *R = f/*ln(*NR*), where *R* is the rate of [*PSI*^*+*^] formation, *f* is the observed frequency of [*PSI*^*+*^] colonies, and *N* is the number of cells in the culture (58, 59).

### AFM Analysis.

The fibril samples were diluted 1:100 for Sup35NM, and 20-μL droplets were deposited on freshly cleaved mica discs (Agar Scientific F7013). After 10-min incubation at room temperature, excess sample was removed by washing with 1 mL of 0.2-µm syringe-filtered mQ H_2_O, and the specimens were then dried under a gentle stream of N_2_(g). For Aβ42 fibrils, samples were diluted 1:10, and 10 μL were deposited on mica disk, let dry at room temperature, washed with 500 μL of mQ H_2_O, and then dried under a gentle stream of N_2_(g). Samples were imaged using a Bruker Multimode AFM with a Nanoscope V controller and a ScanAsyst probe (Silicone nitride tip with nominal tip radius = 2 nm, nominal spring constant 0.4 N/m, and nominal resonant frequency 70 kHz). Images were captured at a resolution of 4.88 nm per pixel scanned. All images were processed using the Nanoscope analysis software (version 1.5, Bruker). The image baseline was flattened using third order baseline correction to remove tilt and bow. Processed image files were opened and analyzed using automated scripts written in Matlab (60).

## Supplementary Material

Supplementary File

## Data Availability

All study data are included in the article and/or *SI Appendix*.
