# Supplementary material for: Amyloid particles facilitate surface-catalyzed cross-seeding by acting as promiscuous nanoparticles
Source: Proc Natl Acad Sci U S A. 2021 Aug 30;118(36):e2104148118. doi: 10.1073/pnas.2104148118 (PMC8433567; doi:10.1073/pnas.2104148118)
Supplement: Supplementary File [file pnas.2104148118.sapp.pdf]

PNAS
www.pnas.org

Supplementary Information for

Amyloid particles facilitate surface-catalyzed cross-seeding by acting as promiscuous nanoparticles

Nadejda Koloteva-Levine[1], Liam D. Aubrey[1], Ricardo Marchante[1], Tracey J. Purton[1], Jennifer R. Hiscock[2], Mick F. Tuite[1] and Wei-Feng Xue[1]*

[1] Kent Fungal Group, School of Biosciences, University of Kent, CT2 7NJ, Canterbury, UK
[2] School of Physical Sciences, University of Kent, CT2 7NJ, Canterbury, UK
* To whom correspondence may be addressed, Email: W.F.Xue@kent.ac.uk

This PDF file includes:

Supplementary text
Figures S1 to S6
Table S1
SI References

Supplementary Information Text

Background theory

A full description of the dynamic mechanism of any amyloid assembly reaction can be formally written using the chemical master equation (1):

$$\frac{d\mathbf{C}}{dt} = \mathbf{k} \cdot \mathbf{C} = \left(\mathbf{k}_{nucleation} + \mathbf{k}_{elongation} + \mathbf{k}_{secondary\,nucleation} + \mathbf{k}_{fragmentation}\right) \cdot \mathbf{C}$$

Eq. S1

In Eq. S1, $\mathbf{C}$ is a column vector that describes the distribution of a discrete set of species involved in the mechanism (e.g. monomers, dimers, trimers, etc.) and their molar concentrations at any time t. The matrix $\mathbf{k}$ represents the rate constants of all possible microscopic reaction steps (e.g. monomers to dimers, dimers back to monomers, dimers to trimers, trimers back to dimers, etc.). Thus, Eq. S1 represents a generic ordinary differential equation (ODE) system consisting of an infinite number of coupled equations, each describing a possible microscopic reaction step. Four processes has been shown to be crucial for amyloid assembly, primary nucleation, growth by elongation at fibril ends, secondary nucleation catalyzed by surfaces of existing amyloid fibrils, and fibril fragmentation. Thus, in Eq. S1, the rate constants in $\mathbf{k}$ can be broken up and grouped to individual terms, each describing a key process. The overall reaction progress of amyloid assembly reactions can be monitored by the amyloid specific fluorescent dye Thioflavin T (ThT), which is sensitive to the mass fraction of monomers in the amyloid state (2). Thus, for the analyses of ThT traces of amyloid assembly reactions from single type of monomers, Eq. S1 can be collapsed into three ODEs:

$$\frac{dM(t)}{dt} = v_{nucleation} + v_{elongation} + v_{secondary\,nucleation}$$

$$\frac{dF(t)}{dt} = v_{nucleation} + v_{secondary\,nucleation} + v_{fragmentation}$$

$$\frac{dm(t)}{dt} = -\frac{dM(t)}{dt}$$

Eq. S2

In Eq. S2, $m(t)$ is the free monomer concentration and $M(t)$ is the concentration of monomers in amyloid fibrils, at any time t. The total monomer concentration is, therefore: $m_{tot} = m(t) + M(t)$. During any amyloid formation reaction, the change in M concentration can be approximately taken to be proportional to the ThT fluorescence signal, and depends on the reaction velocities (v) of primary nucleation, elongation, and secondary nucleation. Primary nucleation velocity depends on the free monomer concentration (m), and can be approximated as $n_c k_n \cdot m(t)^{n_c}$ where n_c is the size of nuclei formed in solution and k_n is the primary nucleation rate constant (3, 4). Secondary nucleation velocity depends on the free monomer concentration and the fibril mass concentration which is proportional to the concentration of monomers in amyloid fibrils (M), and can be approximated as $n_2 k_2 \cdot M \cdot m(t)^{n_2}$ where n_2 is the size of the nuclei formed on the existing fibril surfaces and k_2 is the secondary nucleation rate constant (3, 4). Elongation velocity depends on the free monomer concentration (m) and the particle concentration of fibrils or fibril seeds, F. In addition, the net elongation velocity also depends on the rate of monomers dissociating from the fibril ends. Therefore, the elongation velocity can be expressed as $(k_+ m(t) - k_d) \cdot F(t)$ where k_+ is the elongation rate constant and k_d is the monomer dissociation rate constant. Fragmentation does not contribute to changes in M because the total number of monomers in the amyloid state in an amyloid fibril breaking into two is considered to be the same. $F(t)$ in Eq. S2 is the particle concentration of amyloid fibrils in molar unit, and therefore, this is the seed concentration at any given time t. The change in F concentration depends on the reaction velocities of primary nucleation, secondary nucleation and fragmentation. Elongation at fibril ends does not contribute to changes in F since the growth of fibrils in size through elongation does not lead to an increase in the number of fibrils. The reaction velocity for fragmentation can be assumed to be negligibly

small compared to the other two contributions to changes in F under quiescent experimental conditions, and under conditions where fibril seeds are small in size (5). The contributions from primary and secondary nucleation to changes in F can be approximated in similar manner as in the case of M. Putting all of the considerations above into Eq. S2 yields the following ODE system that can be used to describe experimental ThT traces.

$$\dot{M}(t) = \frac{dM(t)}{dt} = n_c k_n \cdot m(t)^{n_c} + (k_+ m(t) - k_d) \cdot F(t) + n_2 k_2 \cdot M(t) \cdot m(t)^{n_2}$$
$$\dot{F}(t) = \frac{dF(t)}{dt} = k_n \cdot m(t)^{n_c} + k_2 \cdot M(t) \cdot m(t)^{n_2}$$
$$\dot{m}(t) = \frac{dm(t)}{dt} = -\frac{dM(t)}{dt}$$

Eq. S3

For the analyses of ThT traces of heterotypic amyloid assembly reactions from two types of monomers, Eq. S2 can be expanded to include additional three equations for the second monomer type, as well as additional terms compared to Eq. S2 and Eq. S3 to account for the contributions of cross-interactions, e.g. surface catalyzed heterogeneous nucleation, cross-elongation at fibril ends, and co-aggregation. However, for cross-seeded reactions, since the particle concentration and the monomer equivalent concentration of the heterologous seeds is likely to remain relatively constant due to negligible fragmentation and monomer dissociation under quiescent growth conditions, terms describing co-aggregation can be assumed to be negligible. Eq. S3 can then be modified to include cross-seeding due to elongation of heterologous seeds or surface catalyzed nucleation on heterologous seeds:

$$\dot{M}(t) = n_c k_n \cdot m(t)^{n_c} + (k_+ m(t) - k_d) \cdot F(t) + n_2 k_2 \cdot M(t) \cdot m(t)^{n_2} + k_{+,II} m(t) \cdot F_{II}(t) + n_{2,II} k_{2,II} \cdot M_{II}(t) \cdot m(t)^{n_{2,II}}$$
$$\dot{M}_{II}(t) = 0$$
$$\dot{F}(t) = k_n \cdot m(t)^{n_c} + k_2 \cdot M(t) \cdot m(t)^{n_2} + k_{+,II} m(t) \cdot F_{II}(t) + k_{2,II} \cdot M_{II}(t) \cdot m(t)^{n_{2,II}}$$
$$\dot{F}_{II}(t) = -k_{+,II} m(t) \cdot F_{II}(t)$$
$$\dot{m}(t) = -\dot{M}(t)$$
$$\dot{m}_{II}(t) = 0$$

Eq. S4

In Eq. S4, the index 'II' denotes concentrations and rate constants arising due to the heterologous seeds added. Interestingly, the monomer equivalent concentration of heterologous seeds Eq. S4 can be solved numerically with given initial concentrations of monomers and seeds, and analytical or numerical solutions can be fit globally to experimental data series (e.g. (6)) to validate model predictions and to extract information regarding kinetic rate constants. Here, the equation system Eq. S4 was solved numerically for relevant sets of initial concentrations and globally fit to ThT traces of seeded amyloid forming reactions (Supplementary Figure S4) as previously described (2). Experiments with specific initial concentrations of seeds were also designed to allow isolation of the elongation and surface nucleation terms in Eq. S3 and S4 to resolve the heterologous cross-seeding mechanism as detailed below.

An amyloid assembly reaction trace reported by ThT and normalized to the upper stationary baseline is sensitive to the mass fraction of monomers in the amyloid state (2). Thus, the normalized ThT signal intensity, I, can be assumed to be proportional to the following:

$$I(t) = \frac{M(t) - M(t=0)}{M(t \rightarrow \infty) - M(t=0)}$$

Eq. S5

To isolate the reaction rates that account for elongation and enable its comparison with the rates of surface catalyzed nucleation (i.e. secondary nucleation for homologous seeds and

heterogeneous nucleation for heterologous seeds), the initial reaction rates can be evaluated. Using Eq. S3 and S5, the change of normalized ThT signal as function of time is:

$$\dot{I}(t) = \frac{dI(t)}{dt} = \frac{d}{dt}\left(\frac{M(t) - M(t=0)}{M(t\to\infty) - M(t=0)}\right) = \frac{d}{dt}\left(\frac{M(t)}{M(t\to\infty) - M(t=0)} - \frac{M(t=0)}{M(t\to\infty) - M(t=0)}\right)$$
$$= \frac{\dot{M}(t)}{M(t\to\infty) - M(t=0)}$$

Eq. S6

The initial slope of the normalized ThT signal, $\dot{I}_0 = \dot{I}(t=0)$, is then for a self-seeded reaction:

$$\dot{I}_0 = \dot{I}(t=0) = \frac{\dot{M}(t=0)}{M(t\to\infty) - M_0} = \frac{n_c k_n \cdot {m_0}^{n_c} + (k_+ m_0 - k_d)\cdot F_0 + n_2 k_2 \cdot M_0 \cdot {m_0}^{n_2}}{M(t\to\infty) - M_0}$$

Eq. S7

In Eq. S7, m_0 , M_0 and F_0 are the concentration of free monomers, the concentration of monomers in the seeds added, and the particle concentration of seeds added, at the beginning of a reaction when $t = 0s$, respectively. If the monomer dissociation rate constant k_d is negligibly small compared with the elongation rate constant k_+, as commonly the case for amyloid assembly under their normal growth conditions (4), Equation S7 can be further simplified:

$$k_0 = \dot{I}_0 \approx \frac{n_c k_n \cdot {m_0}^{n_c} + k_+ m_0 \cdot F_0 + n_2 k_2 \cdot M_0 \cdot {m_0}^{n_2}}{m_{tot} - M_0} = \frac{n_c k_n \cdot {m_0}^{n_c} + k_+ m_0 \cdot F_0 + n_2 k_2 \cdot M_0 \cdot {m_0}^{n_2}}{m_0}$$
$$= n_c k_n \cdot {m_0}^{n_c - 1} + k_+ \cdot F_0 + n_2 k_2 \cdot M_0 \cdot {m_0}^{n_2 - 1}$$

Eq. S8

Analogously, for a cross-seeded reaction, using Eq. S4 and S5 and inputting that the concentration of homologous seeds, M_0 and F_0, is zero:

$$k_0 = \dot{I}_0 \approx \frac{n_c k_n \cdot {m_0}^{n_c} + k_+ m_0 \cdot F_0 + n_2 k_2 \cdot M_0 \cdot {m_0}^{n_2} + k_{+,II} m_0 \cdot F_{II}(t) + n_{2,II} k_{2,II} \cdot M_{0,II} \cdot {m_0}^{n_{2,II}}}{m_{tot} - M_0}$$
$$= \frac{n_c k_n \cdot {m_0}^{n_c} + k_{+,II} m_0 \cdot F_{0,II} + n_{2,II} k_{2,II} \cdot M_{0,II} \cdot {m_0}^{n_{2,II}}}{m_0}$$
$$= n_c k_n \cdot {m_0}^{n_c - 1} + k_{+,II} \cdot F_{0,II} + n_{2,II} k_{2,II} \cdot M_{0,II} \cdot {m_0}^{n_{2,II} - 1}$$

Eq. S9

As seen above, apart from the difference in the identity of the seeds and the rate constants associated with their reactions, Eq. S8 and S9 are identical. Thus, the initial slope of seeded amyloid formation reactions monitored by ThT, k_0, can be expressed using Eq. S8 for any single type of seeds. Eq S8 (and S9) is, therefore, particularly useful for analyzing self-seeded as well as cross-seeded amyloid assembly reactions because M_0 and F_0 can represent the concentrations of any type of homologous or heterologous seeds added, and the initial seed concentrations can be easily varied experimentally. The initial particle concentration F_0 is linked to the monomer equivalent concentration M_0 in added seeds through the length distribution of the seeds added (1):

$$F = \frac{M}{N \cdot L}$$

Eq. S10

In Eq S10, N is the number of monomers per unit length of fibrils and L is the average length of the fibrils, both parameters can be estimated by imaging (1, 5). In the case where the same stock of seeds are added to seeded reactions, the length distribution of the seeds added will be the same and the initial slope k_0 is linearly proportional to M_0, with contributions from both elongation and surface catalyzed nucleation to the proportionality constant. For self-seeded reactions, the

elongation rate dominate over secondary nucleation, but for cross-seeded reaction, the relative contributions between elongation and surface-catalyzed nucleation (second and third term in Eq. S8, respectively) may vary depending on the precise protein pair. In the case where the same seeds sonicated to different extents are added to seeded reactions, the monomer equivalent concentration M_0 will stay constant but the initial particle concentration F_0 will vary since the average length of the seeds L will change. In that case, as seen in Eq. S8, the initial slope k_0 will only be linearly proportional to F_0 (with a non-zero proportionality constant) due to elongation. Thus, if a constant k_0 that does not vary with changes in F_0 is observed then that will mean elongation rate is negligibly small compared to the surface nucleation based contributions such as surface catalyzed nucleation, leading to prediction II (Figure 1c).

Supplementary Figures

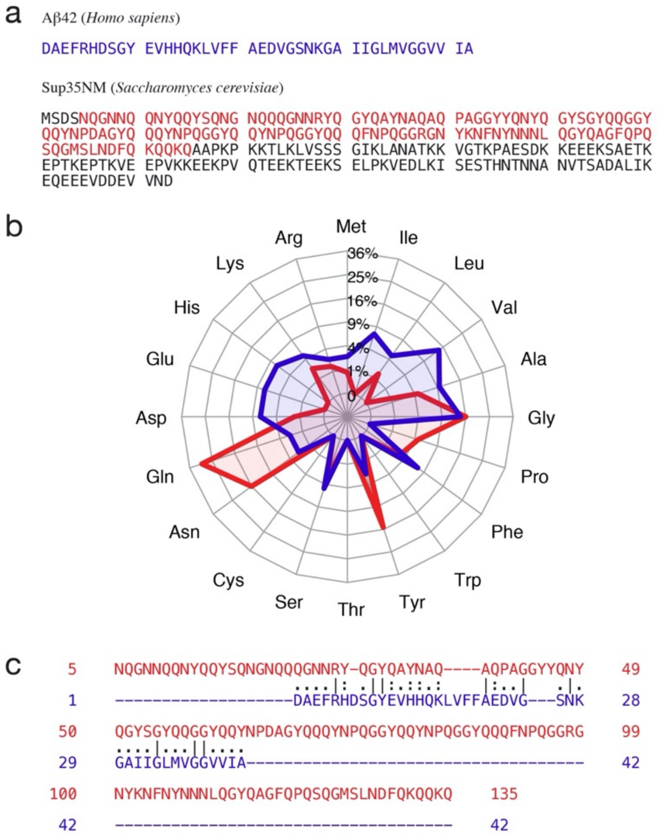

Fig. S1. Aβ42 and Sup35NM have dissimilar amino acid compositions and low sequence similarities. (a) The primary amino acid sequences of Aβ42 and Sup35NM. For Sup35NM, the prion forming domain is highlighted in red. (b) The amino acid composition of Aβ42 and the prion domain of Sup35NM visualized in a web chart. (c) Global pairwise sequence alignment of Aβ42 and the prion domain of Sup35NM using the Needle algorithm with standard parameters. The two sequences share 6.6% identity (lines).

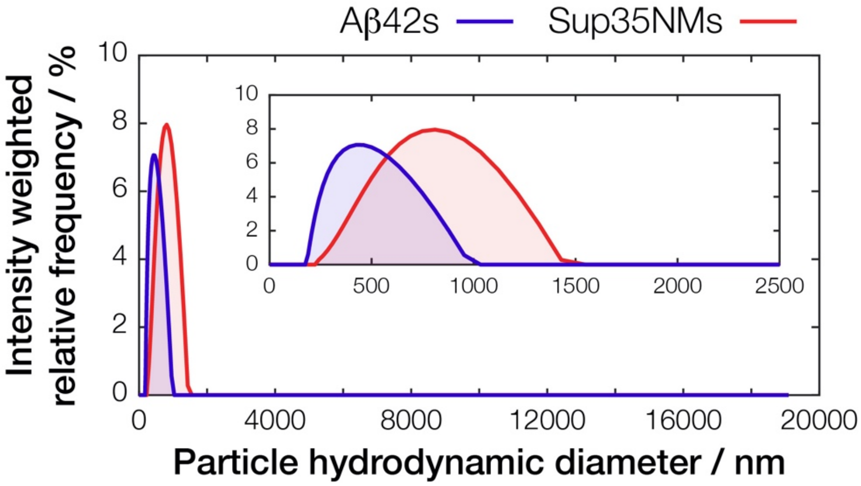

Fig. S2. DLS characterisation of Aβ42s and Sup35NMs samples. Typical DLS traces collected at 25 °C of the same samples as those seen in Figure 2b at a monomer equivalent concentration of 1 μM are shown. The plot with an extended x-axis as well as an expanded view (inset) are shown for clarity.

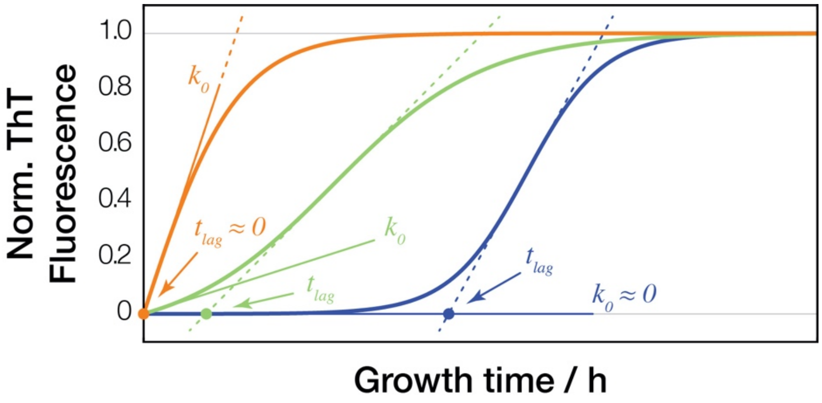

Fig. S3. Schematic illustration of the methods used to extract t_{lag} and k_0 values from kinetics traces of amyloid formation monitored by ThT fluorescence. Three typical example cases are shown together with indications of their t_{lag} and k_0 values.

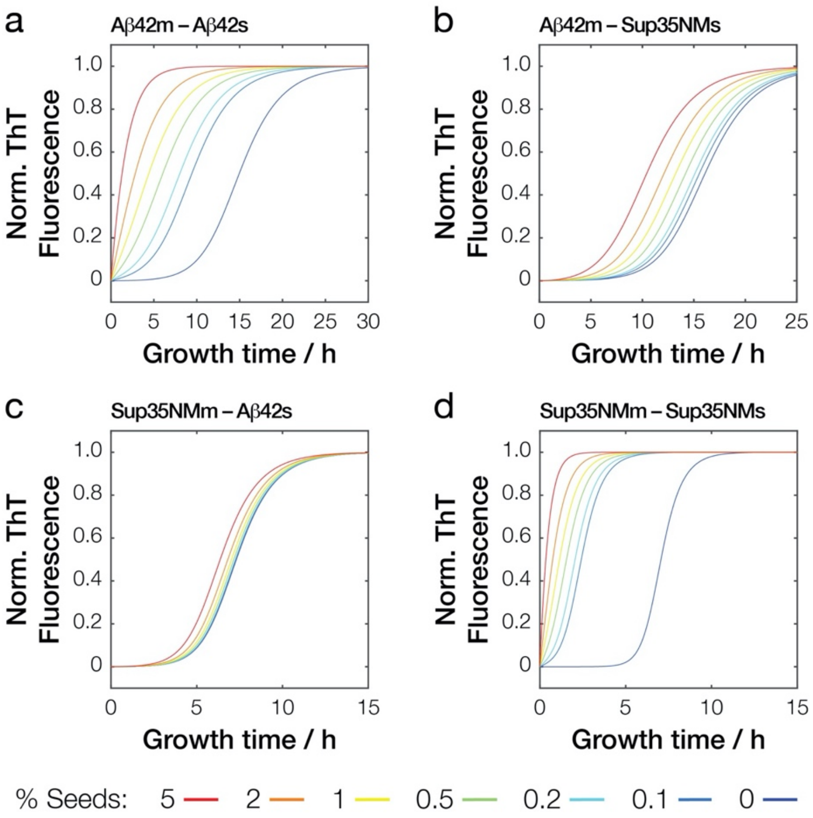

Fig. S4. Best fit kinetics traces of seeded amyloid formation from global analysis of ThT fluorescence kinetics data. Normalized kinetics traces of (a) monomers of Aβ42 (Aβ42m) self-seeded by Aβ42 seeds (Aβ42s) or (b) by Sup35NM seeds (Sup35NMs), as well as (c) Sup35NM monomers (Sup35NMm) seeded by Aβ42 seeds (Aβ42s) or (d) self-seeded by Sup35NM seeds (Sup35NMs) obtained from global analysis of data shown in Figure 3. Under the experimental conditions used, the self-seeded elongation rate constants (Eq. S4) obtained from the globally fitted models are $4.0 \cdot 10^5$ $M^{-1}s^{-1}$ and $3.5 \cdot 10^4$ $M^{-1}s^{-1}$ for self-seeded Sup35NMm and Aβ42m fibril formation reactions, respectively. These rate constants for elongation are in comparable range to those reported for other amyloid forming systems (e.g. (1, 4)). The cross-seeded surface nucleation rate constants (Eq. S4) are 7.2 $M^{-2}s^{-1}$ and $6.7 \cdot 10^1$ $M^{-2}s^{-1}$ for Sup35NMm – Aβ42s and Aβ42m – Sup35NMs pairs, respectively. These values are two to three orders of magnitude smaller than the secondary nucleation rate constant for Aβ42 amyloid formation (4). All kinetic rate constants used to reproduce these globally fitted traces of seeded amyloid formation are shown in Supplementary Table S1.

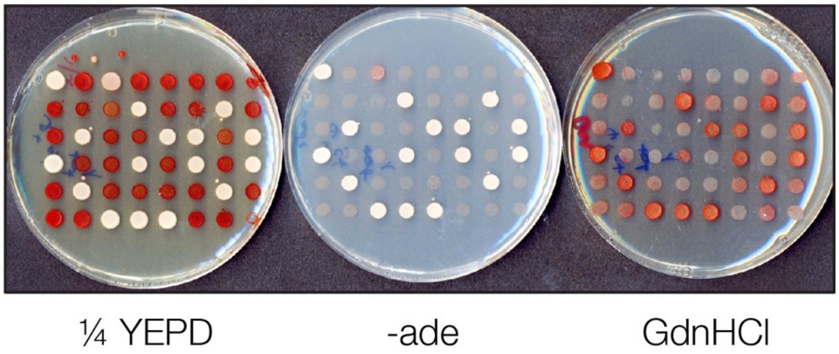

Fig. S5. Yeast cells transfected with a cell extract of positive [*PSI*$^+$] control cells. Positive yeast transfection control where [*PSI*$^+$] conversion is carried out using independent [*PSI*$^+$] yeast cell extracts samples containing *in vivo* formed Sup35 amyloid aggregates.

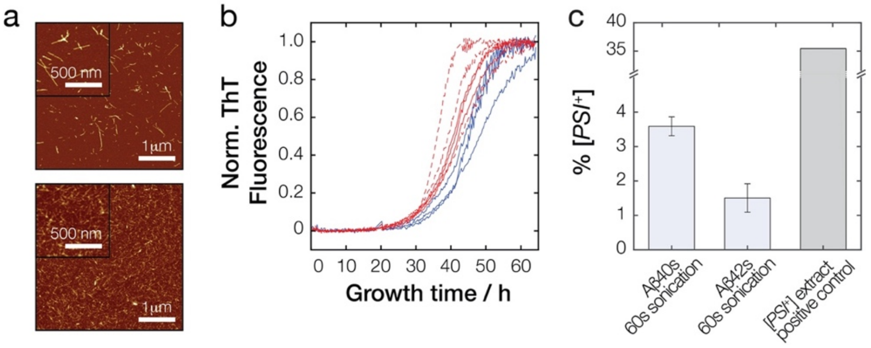

Fig. S6. Aβ40s amyloid seeds is also capable of increasing the rate of Sup35NM amyloid formation *in vitro*, and enhance [*PSI*+] conversion *in vivo* when transfected into yeast cells. (a) AFM images of Aβ40s fibril seeds before (top image) and after 300s of controlled sonication (bottom image). The scale bars indicate 1 μm and the insets show 2 times magnified areas for each image with the scale bars correspond to 500 nm. (b) Normalized traces of non-seeded amyloid formation by 5 μM Sup35NMm monomers (blue traces), or the same concentration of Sup35NMm seeded by 5% (mol/mol) of Aβ40s seeds after 5s (red traces) or 300s (red dashed traces) of sonication. (c) Quantification and comparison of the transfection efficiency displayed by the yeast cells transfected with Aβ40s or Aβ42s seed particles after 60s of controlled sonication, with the light blue bars indicating average values of at least three independent experiments performed on separate days and the error bars represent the standard error of mean. The right dark grey bar indicate the transfection efficiency displayed by the yeast cells transfected with a cell extract of positive [*PSI*+] control (Supplementary Figure S5) for comparison.

Supplementary Table

Table S1. Kinetic parameters obtained from global analysis of ThT fluorescence kinetics data. Kinetic parameters used to reproduce the fitted traces of seeded amyloid formation shown in Supplementary Figure S4 (based on Eq. S4) are shown together with their respective standard error (SE). The parameters n_c, n_2 and $n_{2,II}$ are approximated to be 2 based on the same assumption used in (4). The dissociation rate constant k_d is assumed to be negligible compared to k_+ (Eq. S8 and Eq. S9).

	k_n / $M^{-1}s^{-1}$ (log k_n ± SE)	k_+ / $M^{-1}s^{-1}$ (log k_+ ± SE)	k_2 / $M^{-2}s^{-1}$ (log k_2 ± SE)	$k_{2,II}$ / $M^{-2}s^{-1}$ (log $k_{2,II}$ ± SE)
Aβ42m-Aβ42s	$5.4 \cdot 10^{-6}$ (-5.3 ± 0.4)	$3.5 \cdot 10^{4}$ (4.5 ± 0.1)	$6.8 \cdot 10^{2}$ (2.8 ± 0.3)	–
Aβ42m-Sup35NMs	$4.2 \cdot 10^{-6}$ (-5.4 ± 0.2)	$3.5 \cdot 10^{4}$ (4.5 ± 0.1)	$5.8 \cdot 10^{2}$ (2.8 ± 0.1)	$6.7 \cdot 10^{1}$ (1.8 ± 0.1)
Sup35MNm-Aβ42s	$4.2 \cdot 10^{-6}$ (-5.4 ± 0.9)	$2.6 \cdot 10^{5}$ (5.4 ± 0.3)	$2.8 \cdot 10^{2}$ (2.5± 0.2)	7.2 (0.9 ± 0.4)
Sup35NMm-Sup35NMs	$3.4 \cdot 10^{-7}$ (-6.5 ± 0.8)	$4.0 \cdot 10^{5}$ (5.6 ± 0.2)	$3.3 \cdot 10^{2}$ (2.5 ± 0.5)	–

SI References

1. W.-F. Xue, S. E. Radford, An imaging and systems modeling approach to fibril breakage enables prediction of amyloid behavior. *Biophys J* **105**, 2811-2819 (2013).
2. W.-F. Xue, S. W. Homans, S. E. Radford, Systematic analysis of nucleation-dependent polymerization reveals new insights into the mechanism of amyloid self-assembly. *Proc Natl Acad Sci U S A* **105**, 8926-8931 (2008).
3. S. I. Cohen, M. Vendruscolo, C. M. Dobson, T. P. Knowles, Nucleated polymerization with secondary pathways. II. Determination of self-consistent solutions to growth processes described by non-linear master equations. *J Chem Phys* **135**, 065106 (2011).
4. S. I. A. Cohen *et al.*, Proliferation of amyloid-β42 aggregates occurs through a secondary nucleation mechanism. *Proceedings of the National Academy of Sciences* **110**, 9758-9763 (2013).
5. D. M. Beal *et al.*, The Division of Amyloid Fibrils: Systematic Comparison of Fibril Fragmentation Stability by Linking Theory with Experiments. *iScience* **23**, 101512 (2020).
6. G. Meisl *et al.*, Molecular mechanisms of protein aggregation from global fitting of kinetic models. *Nat Protoc* **11**, 252-272 (2016).
